# Fusions involving BCOR and CREBBP are rare events in infiltrating glioma

**DOI:** 10.1186/s40478-020-00951-4

**Published:** 2020-06-03

**Authors:** David J. Pisapia, Kentaro Ohara, Rohan Bareja, David C. Wilkes, Erika Hissong, Jaclyn A. Croyle, Joon-Hyung Kim, Jad Saab, Theresa Y. MacDonald, Shaham Beg, Catherine O’Reilly, Sarah Kudman, Mark A. Rubin, Olivier Elemento, Andrea Sboner, Jeffrey Greenfield, Juan Miguel Mosquera

**Affiliations:** 1 Department of Pathology and Laboratory Medicine, Weill Cornell Medicine, 1300 York Avenue, New York, NY 10065 USA; 2 Caryl and Israel Englander Institute for Precision Medicine, Weill Cornell Medicine and NewYork-Presbyterian, 413 East 69th Street, New York, NY 10021 USA; 3 Department of Physiology and Biophysics, Weill Cornell Medicine, 1300 York Avenue, New York, NY 10065 USA; 4 Institute for Computational Biomedicine, Weill Cornell Medicine, 1305 York Avenue, New York, NY 10021 USA; 5 Department of Neurological Surgery, Weill Cornell Medicine, 525 East 68 Street, New York, NY 10065 USA

**Keywords:** Infiltrating glioma, BCOR, CREBBP, Fusion

## Abstract

*BCOR* has been recognized as a recurrently altered gene in a subset of pediatric tumors of the central nervous system (CNS). Here, we describe a novel *BCOR-CREBBP* fusion event in a case of pediatric infiltrating astrocytoma and further probe the frequency of related fusion events in CNS tumors. We analyzed biopsy samples taken from a 15-year-old male with an aggressive, unresectable and multifocal infiltrating astrocytoma. We performed RNA sequencing (RNA-seq) and targeted DNA sequencing. In the index case, the fused *BCOR-CREBBP* transcript comprises exons 1–4 of *BCOR* and exon 31 of *CREBBP*. The fused gene thus retains the Bcl6 interaction domain of BCOR while eliminating the domain that has been shown to interact with the polycomb group protein PCGF1. The fusion event was validated by FISH and reverse transcriptase PCR. An additional set of 177 pediatric and adult primary CNS tumors were assessed via FISH for *BCOR* break apart events, all of which were negative. An additional 509 adult lower grade infiltrating gliomas from the publicly available TCGA dataset were screened for *BCOR* or *CREBBP* fusions. In this set, one case was found to harbor a *CREBBP-GOLGA6L2* fusion and one case a *CREBBP-SRRM2* fusion. In a third patient, both *BCOR-L3MBTL2* and *EP300-BCOR* fusions were seen. Of particular interest to this study, *EP300* is a paralog of *CREBBP* and the breakpoint seen involves a similar region of the gene to that of the index case; however, the resultant transcript is predicted to be completely distinct. While this gene fusion may play an oncogenic role through the loss of tumor suppressor functions of BCOR and CREBBP, further screening over larger cohorts and functional validation is needed to determine the degree to which this or similar fusions are recurrent and to elucidate their oncogenic potential.

## Introduction

Fusion transcripts are increasingly recognized as important oncogenic drivers in tumors of the central nervous system (CNS). These include *KIAA1549-BRAF*, typically resulting from tandem duplication and characteristic of pilocytic astrocytoma [19], *C11orf95-RELA* in supratentorial ependymoma [32], and *FGFR-TACC* (e.g. *FGFR3-TACC3*) in a subset of infiltrating gliomas [40], among others. Recurrent fusion transcripts have additionally been identified in pediatric infiltrating gliomas, including those involving the *MYB* and *MYBL1* loci [36]. As tumors of the CNS continue to be profiled using RNA sequencing or other platforms to detect fusion transcripts, it is likely that more fusion driver candidates will be discovered.

BCL6 interacting co-repressor (*BCOR*), a gene whose product has been shown to interact with histone deacetylases and enhances BCL6-mediated transcriptional repression [17], has recently been recognized as recurrently altered in a subset of pediatric tumors of the CNS with embryonal features. These tumors, termed “high grade neuroepithelial tumor with BCOR alteration” (HGNET-BCOR), are characterized in most cases by an internal tandem duplication involving exon 15 of the gene [42]. Fusion transcripts involving the *BCOR* gene have also been described in a diversity of tumors extrinsic to the CNS including clear cell sarcoma of the kidney [37, 48], ossifying fibromyxoid tumors [21], acute promyelocytic leukemia [50], endometrial stromal sarcoma (ESS) [27, 31], adult non-uterine sarcoma [51], and a subset of small blue round cell sarcomas [34, 35, 41].

More recently, *EP300-BCOR* alterations have been described in pediatric gliomas [46]. Herein, we describe a similar fusion event involving *BCOR* and *CREBBP*. CREBBP is a paralogue of EP300 acetytransferase. The identified fusion event has not been previously reported in a pediatric infiltrating glioma, and we further explore the extent to which these genes are altered in a broader set of central nervous system tumors, including adult gliomas.

## Methods

### Next-generation sequencing (NGS)

The Oncomine Comprehensive Assay v3 (OCAv3) (ThermoFisher Scientific) was performed on the IonTorrent™ S5 XL platform, following manufacturer protocols (https://assets.thermofisher.com/TFS-Assets/LSG/manuals/MAN0015885_OncomineComprehensiveAssay_v3_UG.pdf

Last downloaded 12/27/2019). OCAv3 is an amplicon-based targeted assay that enables the detection of relevant SNVs, amplifications, gene fusions, and indels from 161 unique genes (Supplementary Table 1).

### RNA sequencing and fusion confirmation by RT-PCR and sanger sequencing

RNA sequencing (RNA-seq) and data processing was performed as previously described [4, 7]. Briefly, RNA was extracted from frozen material for RNA-seq using the Promega Maxwell 16 MDx instrument (Maxwell 16 LEV simplyRNA Tissue Kit (cat. # AS1280)). Specimens were prepared for RNA sequencing using the TruSeq RNA Library Preparation Kit v2 or riboZero as previously described [4]. RNA integrity was verified using the Agilent Bioanalyzer 2100 (Agilent Technologies). cDNA was synthesized from total RNA using Superscript III (Invitrogen). Sequencing was then performed on GAII, HiSeq 2000, or HiSeq 2500 as paired-ends [4, 7]. All reads were independently aligned with STAR_2.4.0f1 [11] for sequence alignment against the human genome sequence build hg19, downloaded via the UCSC genome browser http://hgdownload.soe.ucsc.edu/goldenPath/hg19/bigZips/, and SAMTOOLS v0.1.19 [25] for sorting and indexing reads. Cufflinks (2.0.2) was used to estimate the expression values (FPKMS), and GENCODE v19 GTF file for annotation [9, 47]. For fusion analysis, we used STAR-fusion (STAR-Fusion_v0.5.1), and FusionSeq (v0.7.2) [15, 38] on publically available RNAseq data available from the TCGA Research Network lower grade glioma cohort. Fusions with significant support of junction reads and spanning pairs were then selected for manual review.

For *BCOR-CREBBP* gene fusion analysis, PCR was performed using custom PCR primers designed to amplify short (approximately 200–400 bp) regions. A human gDNA control sample was run in parallel to confirm successful PCR and end-sequencing was performed using PCR primers. After enzymatic purification, sequencing was achieved through BigDye Terminator Cycle Sequencing. Data analysis was performed with DNASTAR Lasergene12 software.

### Fluorescence in-situ hybridization (FISH)

5 m-thick formalin-fixed paraffin-embedded (FFPE) tissue sections were cut for FISH analysis, either as representative whole slides of individual cases, or 3 representative 1 mm tissue cores per case integrated into tissue microarrays. *BCOR* break apart was validated using dual color FISH probes (RP11-973F20 BAC clone labeled red; RP11-1082P20 labeled green). *BCOR* break apart was determined as one individual green signal and one individual red signal, per nucleus. *CREBBP* break apart was validated using dual color FISH probes (RP11-95P2 BAC clone labeled red; RP11-433P17 labeled green). *CREBBP* break apart was determined as one individual green signal, one individual red signal, and one individual green and red signal overlapping, per nucleus. *BCOR-CREBBP* fusion was determined using dual color FISH probes (BAC clone RP11-1082P20 labeled red; RP11- RP11-433P17 labeled green). Fusion was measured as one individual green signal and one individual green and red signal overlapping, per nucleus. Prior to use, all clones were validated on metaphase spreads. A minimum of 100 nuclei were observed per case using a fluorescence microscope (Olympus BX51; Olympus Optical, Tokyo, Japan). Cytovision and Fiji software were used for imaging.

### Immunohistochemistry

For BCOR staining, staining was performed at the Mayo Clinic Laboratories in Rochester, MN on an FFPE 4 μm-thick section from the index tumor case. A commercially available antibody (Santa Cruz C10 monoclonal antibody) was used at a dilution of 1:250. Positive control tissue comprised a FFPE tissue core of an Ewing-like sarcoma with BCOR fusion.

### Gene set enrichment analysis

We calculated z-scores comparing the index case with 82 infiltrating glioma samples over 67 patients (x- mean/sd), from expression values. The z-scores were used as a metric to rank the genes in the sample. The hypergeometric test and Gene Set Enrichment Analysis (GSEA) [43] was used to identify enriched signatures using the different pathways collection in the online MSigDB database (https://www.gsea-msigdb.org/gsea/msigdb/index.jsp). We used the GSEA pre-ranked method which takes the ranked gene list as an input. The infiltrating glioma samples in the comparison group included 8 pediatric high grade gliomas (including two diffuse midline gliomas), 8 IDH-mutated infiltrating astrocytomas, 9 oligodendrogliomas, and 42 IDH-wildtype infiltrating astrocytomas of which 35 had conventional histological features of glioblastoma.

## Results

**Fig. 1:**
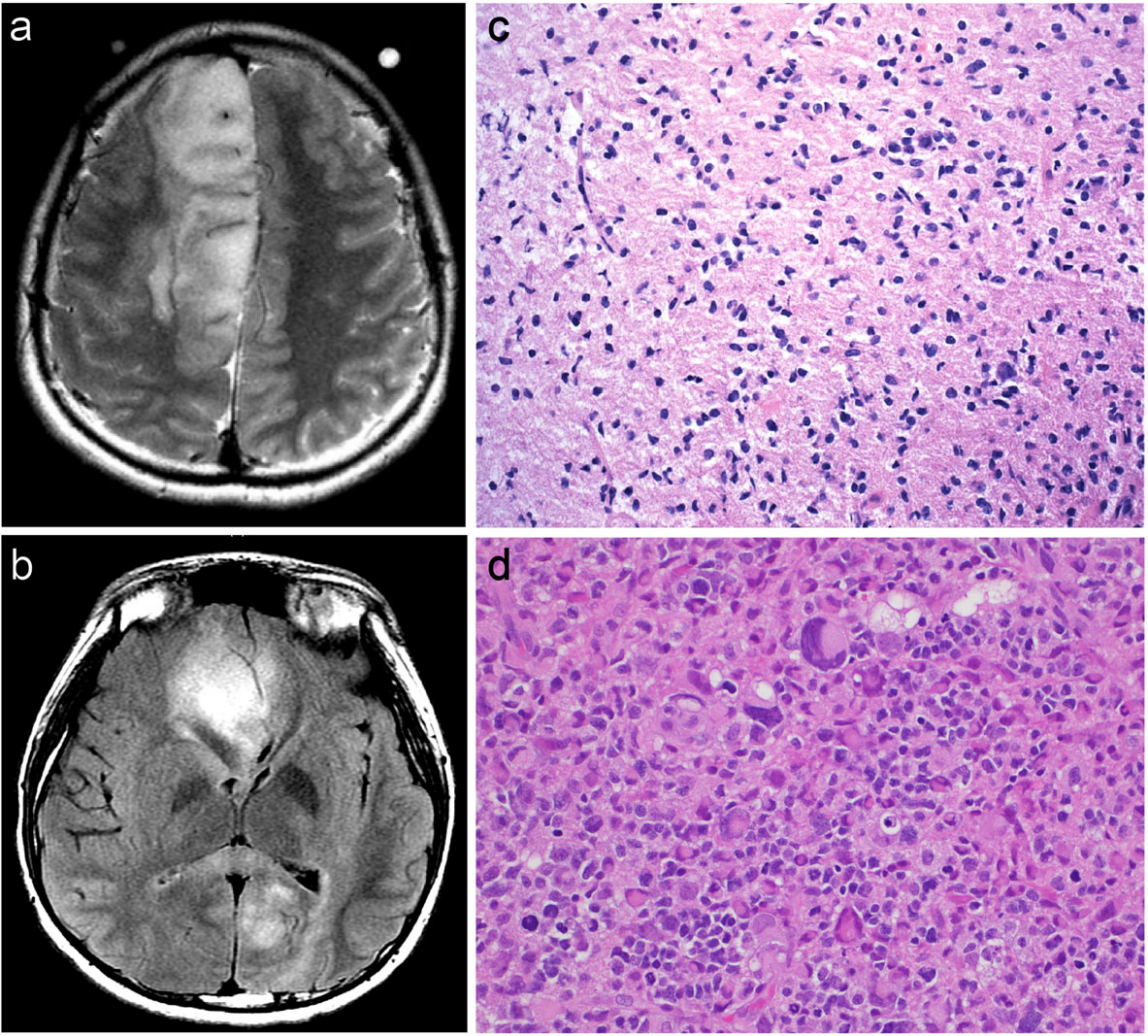
Radiological and histological characteristics of the index case. Preoperative brain MRIs for the primary tumor demonstrated a mass involving the right frontal lobe as well as the left occipital and temporal lobes (**a**, **b**). Representative histology of the primary tumor shows a diffusely infiltrating astrocytoma with predominantly lower grade features (**c**). Re-resection material met histologic criteria for glioblastoma (**d**)

A 15-year-old previously healthy boy presented with new onset seizures. Magnetic resonance imaging (MRI) demonstrated a non-contrast enhancing, expansile mass involving the right frontal, left temporal and left occipital lobes, consistent with an infiltrating glioma and demonstrating a pattern of disease spread historically referred to as “gliomatosis cerebri” (Fig. 1a, b). Right frontal craniotomy and biopsy was performed followed by adjuvant radiotherapy with concomitant temozolomide. Postoperative MRI performed 18 months later revealed progression of disease with a new area of enhancement involving the right parietal lobe. The patient then underwent partial tumor debulking and adjuvant radiotherapy and chemotherapy (temozolomide, bevacizumab and carboplatin). The patient continued to deteriorate clinically and treatment was discontinued 27 months following initial biopsy.

### Molecular characterization of genomic alterations

Histological evaluation of the initial biopsy tissue showed a diffusely infiltrative astrocytoma with predominantly low-grade features (Fig. 1c). A targeted next generation sequencing panel (Oncomine®v3) revealed truncating mutations in *NF1* and *ARID1A*. A *TERT* promoter mutation was also present (Table 1). While the panel did not assess for *ATRX* mutations, immunohistochemical analysis demonstrated loss of expression of ATRX. In addition, targeted PCR followed by Sanger sequencing and immunohistochemistry was conducted to rule out mutations of *H3F3A* at codons 27 and 34. If current recommendations for the adult setting were to be applied, the presence of *TERT* promoter mutation in combination with an absence of *IDH1/IDH2* mutation would be compatible with a diagnosis of *diffuse astrocytic glioma, with molecular features of glioblastoma, WHO grade IV* [5]. Following chemoradiation, the patient underwent re-resection of disease for recurrence and the histological features, including markedly increased pleomorphism and cellularity (Fig. 1d) as well as necrosis, were at this time compatible with a histological diagnosis of glioblastoma (Fig. 1d). Tissue from the re-resection material was not available for molecular analysis.

**Table 1:** Summary of molecular data interrogated by Oncomine, PCR and immunohistochemistry

POSITIVE CALLS	PERTINENT NEGATIVES
*ARID1A* p.Asp204fs	mutations in *IDH1*, *IDH2*
*TERT* promoter mutation c.-124C > T	H3 K27M and G34 by IHC and PCR/Sanger
*NF1* p.Trp696Ter	*EGFR* amplification
ATRX loss of expression by IHC	Remaining Oncomine Panel Targets^a^

Next generation sequencing using the Oncomine® panel v3. Positive calls are listed in the left column while selected pertinent negatives are listed in the right column

^a^See Supplementary Table 1 for a complete list of genes interrogated by the Oncomine panel

### Analysis of *BCOR-CREBBP* fusion transcript

**Fig. 2:**
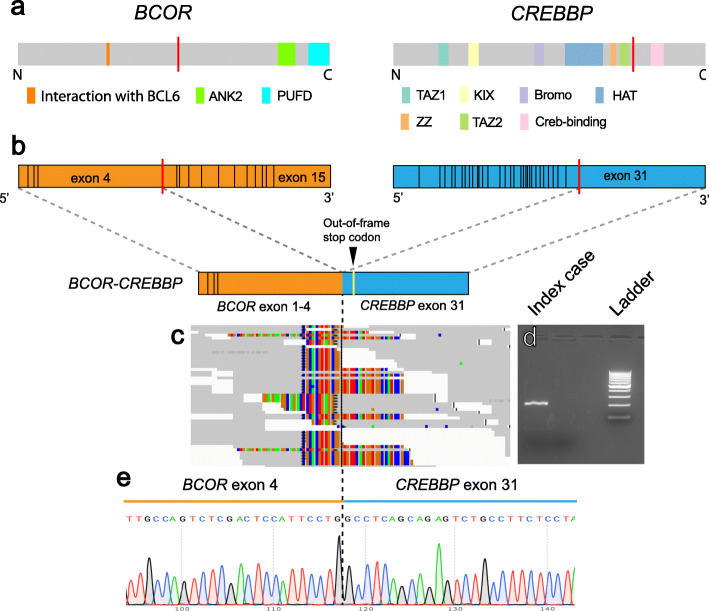
Description and validation of BCOR-CREBBP fusion product. Structure and functional domains of BCOR and CREBBP with the red line indicating the fusion point (**a**). The detected fusion joins exon 4 of *BCOR* on chromosome X with exon 31 of *CREBBP* on chromosome 16 (**b**). RNA sequencing demonstrated multiple reads in support of the fusion transcript (**c**). RT-PCR using primers for *CREBBP* and *BCOR* demonstrates a robust PCR product (**d**). Sanger sequencing confirmed the chimeric DNA transcript, with the black dashed line indicating the fusion point (**e**)

Frozen tissue from the initial biopsy material was utilized for RNA-seq followed by computational analysis using FusionSeq. FusionSeq nominated a *BCOR-CREBBP* fusion event with supporting evidence including 18 junction reads and 33 spanning fragments (Fig. 2b and c). The fusion product comprises exons 1–4 of *BCOR* (with the break occurring at codon 901 in exon 4 at position chrX:39931896) and exon 31 of *CREBBP* (with the break occurring at codon 1877 at position chr16:3779417). The *BCOR-CREBBP* fusion was predicted to be out-of-frame, creating a premature stop codon within the CREBBP segment at codon 1965. The reciprocal *CREBBP-BCOR* fusion transcript was not detected by FusionSeq analysis.

**Fig. 3:**
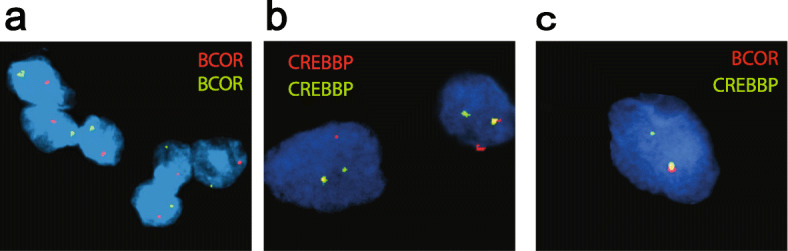
Fluorescent in situ hybridization (FISH) assays for BCOR and CREBBP in the index case. Break-apart green and red signals for *BCOR* (**a**) and *CREBBP* (**b**) demonstrate gene rearrangement at the break points. For *BCOR*, only one allele is present, consistent with a single X chromosome in this male patient. Fusion FISH assay shows the overlapping red and green signals in one allele (yellow signal), confirming *BCOR*-*CREBBP* fusion (**c**)

Further validation was performed by reverse transcription followed by PCR amplification of the putative fused transcript, including the breakpoint (Fig. 2d). Sanger sequencing of the amplified product further confirmed the presence of the breakpoint detected by RNA-seq (Fig. 2e). Disruption of the *BCOR* locus was additionally demonstrated via FISH using a break-apart strategy and probes recognizing loci 3′ and 5′ to the breakpoints of *BCOR* on the X chromosome and *CREBBP* on chromosome 16 (Fig. 3a, b). A fusion FISH strategy was also used to confirm colocalization of *BCOR* and *CREBBP* (Fig. 3c).

Due to the unavailability of residual frozen tissue, we were not able to perform Western blot analysis in an effort to detect a putative protein product. Immunohistochemistry was performed on FFPE tissue sections with an antibody developed against amino acids 1–300 of the BCOR protein; however, we did not detect any labeling in tumor cells in contrast to control tissue of an Ewing-like sarcoma harboring a BCOR fusion (Supplementary Fig. 1). While, this result could be due to failure of translation within tumor cells, we cannot exclude the possibility that the antigenic epitope is masked in the putative altered fused protein in our case, or that the staining protocol was suboptimal for this tumor type.

#### Fusions involving BCOR or CREBBP are rare events in adult and pediatric brain tumors

To explore whether the detected BCOR fusion is a recurrent event in primary brain tumors, break apart FISH for the *BCOR* locus was performed on whole slides or tissue microarrays comprising a diversity of central nervous system neoplasms (Table 2). Out of 177 additional screened cases, zero cases screened by FISH demonstrated evidence of a BCOR fusion event.

**Table 2:** Additional cases screened by break apart FISH for the *BCOR* locus

Diagnosis	Number of cases
Glioblastoma, adult	94
Oligodendroglioma, adult	19
Lower grade infiltrating astrocytoma, adult	17
Pilocytic astrocytoma	11
Ganglioglioma	6
Infiltrating glioma, pediatric	6
Medulloblastoma	6
Ependymoma	5
Meningioma	4
Pilomyxoid astrocytoma	4
Subependymal giant cell astrocytoma	2
Atypical teratoid/rhabdoid tumor	1
CNS Embryonal tumor, NOS	1
Pleomorphic xanthoastrocytoma	1
**Total**	**177**
Positive cases for *BCOR* break-apart signals	0

This table includes a list and number of tumor types screened via FISH for the presence of a break apart event involving the BCOR locus, using tissue microarrays

**Fig. 4:**
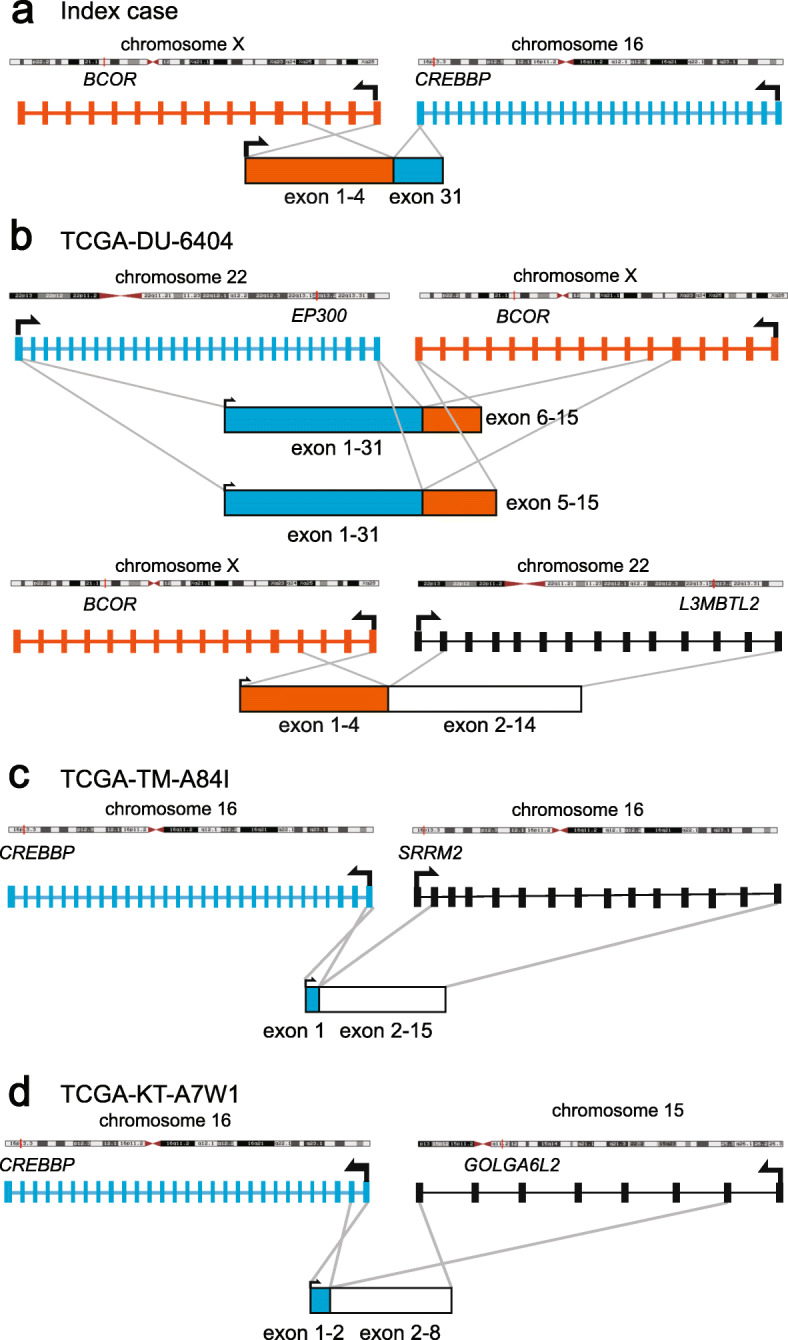
Fusion proteins detected in TCGA cohort with genes relevant to this study. *BCOR-CREBBP* fusion product in the index case (**a**). TCGA-DU-6404 is a 24-year-old female with IDH-wildtype high grade glioma harbored two fusions involving *BCOR*, namely *BCOR-L3MBTL2* and *BCOR-EP300* (**b**). TCGA-TM-A84I is a 30-year-old with IDH-mutant anaplastic astrocytoma harbored a *CREBBP-SRRM2* fusion (**c**). TCGA-KT-A7W1 is 45-year-old with IDH-wildtype anaplastic astrocytoma harbored a *CREBBP-GOLGA6L2* fusion (**d**)

To further screen a larger subset of infiltrating gliomas, analysis of RNA-seq data from The Cancer Genome Atlas (TCGA) cohort of lower grade glioma patients (*n* = 509) was also conducted. The analysis yielded four fusion transcripts relevant to the current study (Fig. 4). Specifically, a 24-year-old female with an IDH-wildtype high grade glioma harbored two fusions involving *BCOR*, namely *BCOR-L3MBTL2* as well as *EP300-BCOR*, the latter with two distinct breakpoints detected (Fig. 4b); a 30-year-old with anaplastic astrocytoma, IDH-mutant, harbored a *CREBBP-SRRM2* fusion (Fig. 4c); and a 45-year-old with anaplastic astrocytoma, IDH-wildtype, harbored a *CREBBP-GOLGA6L2* fusion (Fig. 4d).

Furthermore, we compared the *BCOR-CREBBP* fusion product in the present case to that of previously reported chimeric transcripts involving *BCOR* rearrangements with *CREBBP* or *EP300* in ESS and pediatric gliomas [25, 42]. The previously reported events include *BCOR-CREBBP* and *CREBBP-BCOR* fusions in ESS, and *EP300-BCOR* fusions in 3 cases of pediatric glioma. The extent of the *BCOR* segment of the chimeric transcripts was variable among the reported cases (Supplementary Fig. 2). In the majority, exons 1–30 of CREBBP/EP300 were present in the resultant transcript with one case of ESS showing the inverse, with only exon 31 included, similar to what is seen in the present case (Supplementary Fig. 2).

### Gene set enrichment analysis of the index case

To probe potential downstream biological consequences of BCOR and/or CREBBP alteration, we compared the transcriptional profile of our index case to that of a cohort of 82 samples (over 67 patients) of adult and pediatric infiltrating gliomas for which we had also performed RNAseq. Unbiased gene set rich enrichment analysis revealed two gene sets, among 50 predefined HALLMARK gene sets [26], that demonstrated a nominal *p* value for enrichment of < 0.05 and a false discovery rate of < 0.05 [43]. These gene sets included those relating to oxidative phosphorylation and targets regulated by MYC (Supplemental Fig. 3). Interestingly there is precedent in the cancer literature for MYC pathway activation in the context of BCOR loss in T-cell lymphoblastic leukemia and lymphomas [24, 44].

## Discussion

Genetic alterations resulting in the generation of chimeric fusion transcripts are increasingly recognized as driving events in the oncogenic cascade. Gene fusions have been described in a diversity of CNS tumors similar to other tumor families, including hematopoietic neoplasms and sarcomas. For example, a tandem duplication event linking *BRAF* to a nearby gene, *KIAA1549*, is a recurrent event seen in a majority of pilocytic astrocytomas and represents the predominant neoplastic driver in such cases. Certainly, fusion transcripts have also been described not as singular events but in the setting of multiple other well-described oncogenic events. For example, those infiltrating gliomas with *FGFR-TACC* fusions may present with other oncogenic alterations including *CDKN2A* loss, *CDK4* amplification, *MDM2* amplification and/or *TERT* promoter mutations [10]. In the present case, while well-characterized genes including *NF1* and *ARID1A* were detected using a targeted DNA sequencing panel, analysis of RNA-seq data additionally revealed a *BCOR-CREBBP* fusion event with oncogenic potential that was validated by several other modalities. FISH results suggest this gene fusion results from chromosomal translocation.

Considering the domains represented in the resultant putative BCOR-CREBBP fusion protein, it is possible that this gene fusion has an oncogenic role. BCOR interacts with polycomb group ring finger 1 (PCGF1) through the PCGF Ub-like fold discriminator domain (PUFD) at the C-terminus of BCOR, and is a constituent of the polycomb repressive complex 1.1 (PRC1.1), which is involved in the control of cell differentiation including by the regulation of histone methylation marks [20]. Recent in vivo studies suggest that the PUFD domain is essential for a tumor suppressor function of BCOR and that loss of BCOR promotes leukemogenesis [22, 45]. Moreover, next-generation sequencing studies have revealed various *BCOR* alterations in a broad range of neoplastic diseases [2, 6, 29, 39]. In CNS tumors, loss of function *BCOR* mutations (e.g., nonsense, frameshift, splice sites and deletions) have been described in medulloblastoma, high-grade pediatric gliomas and astroblastomas [2]. Given that the *BCOR* component of the fusion transcript in the present case was truncated from the middle of exon 4 and consequently lacks a PUFD domain, the gene fusion product may be oncogenic in part from a loss of tumor suppressor function of BCOR. Moreover, since the *BCOR* gene is located on the X-chromosome, the *BCOR* gene in the index case is present as only one allele and the fusion event would lead to a complete loss of putative tumor-suppressor activity mediated by the PUFD. Another possibility is that the residual BCOR segment fails to undergo translation at all, as evidenced by the lack of immunohistochemical staining in tumor cells. In this scenario, one possible mechanism underlying this putative oncogenic activity would be through upregulation of genes targeted by MYC; indeed, our transcriptomic analysis reveals enrichment of genes targeted by MYC, in a manner analogous to what has previously been reported in T cell lymphoblastic leukemia and lymphomas that have undergone loss of BCOR [24, 44]. Transcriptomic analysis also revealed upregulation of genes involved in oxidative phosphorylation, which may reveal fundamental shifts in metabolic pathway utilization, exposing potential vulnerabilities in tumors harboring this alteration.

Similar to *BCOR*, the fusion event involving *CREBBP* (cAMP-response element binding protein-binding protein) potentially promotes gliomagenesis via disruption of its tumor suppressor function. CREBBP acts as a chromatin modifier with acetyltransferase activity and is implicated in the transcriptional regulation of both developmental and neoplastic processes [13, 49]. In the brain, this gene and its paralog, *EP300*, are altered in patients with Rubinstein-Taybi syndrome, a rare sporadic neurodevelopmental disorder characterized by neurocognitive deficits, autism-spectrum type behaviors and gross anatomical abnormalities including facial dysmorphism [16]. While the precise function of CREBBP in tumor biology remains largely unknown, several studies have reported that CREBBP and its closely related paralog EP300 behave as haploinsufficient tumor suppressors [33, 53]. In cancer, *CREBBP*/*EP300* is targeted by both mutations and structural alterations. For example, recent studies demonstrated that somatic inactivating mutations of the histone-acetyltransferase (HAT) domain of *CREBBP*/*EP300* impair its acetyltransferase activity in certain types of non-Hodgkin B-cell lymphoma and bladder cancer [12, 33]. In addition, previous data have detected loss of heterozygosity at the *EP300* or *CREBBP* loci in colorectal, gastric, ovarian, and hepatocellular carcinomas [28]. Given that the *CREBBP* component of the fusion transcript in our case retains only exon 31 and consequently lacks most of the functional domains, loss of CREBBP through this gene fusion potentially promotes gliomagenesis. In contrast, several studies suggest that CREBBP/EP300 can also mediate pro-oncogenic functions in some cell types [3]. Further experimental studies would be required in order to clarify the biological role of CREBBP/EP300 in gliomagenesis.

As discussed above, the fusion event in the present case potentially exerts oncogenic activity via the loss of both BCOR and CREBBP. While the pathogenic impact of most reported gene fusions is gain-of-function, such as constitutive kinase activation and abnormal activity of transcriptional factors, fusions resulting in loss of function of tumor suppressors have been identified as well [23, 30]. The *APC-COMMD10* fusion in colorectal cancer is one example wherein fusion-mediated truncation leads to a loss-of-function of tumor suppressors. In particular, Choi et al. suggested that the lack of functional domains in APC resulting from the *APC-COMMD10* gene fusion can lead to tumorigenesis [8] where loss of APC is known to be a critical event in the development of colon cancer [52]. Another example is *RUNX1*-chromosome 9 fusion, which potentially contributes to disease progression of myeloproliferative syndrome through haploinsufficiency of *RUNX1* [1].

Providing further support for the potential oncogenicity of this fusion is the recent discovery of similar fusions in two completely unrelated tumor types. A fusion involving CREBBP with BCORL1 has been described in ossifying fibromyxoid tumors, with a similar breakpoint region to that seen in our case, though with a distinct predicted fusion transcript that preserves the HAT domain of CREBBP [21]. In addition, in a recently published series of supratentorial ependymoma, a single case demonstrated a fusion product between EP300 and BCORL1, also with a similar breakpoint region exon 31 of EP300 and exon 4 of BCORL1, though here too the authors predicted the fusion transcript would have preserved most functional domains in both proteins [14].

To examine the frequency of *BCOR-CREBBP* fusions in CNS tumors more broadly, we analyzed RNA-seq data from 509 cases of lower grade infiltrating gliomas available through the TCGA and performed break apart FISH for the BCOR locus for an additional 177 adult and pediatric brain tumors. We did not find additional cases with the same *BCOR-CREBBP* fusion from these additional analyses, indicating that this fusion gene is likely a rare event in CNS tumors. However, two independent fusion events involving *BCOR* were found in one TCGA case, *BCOR-L3MBTL2* and *EP300-BCOR*. In that case, the breakpoints seen in *BCOR* involve a similar region of the gene to those in the present case, and the *BCOR-L3MBTL2* fusion transcript detected is predicted to contain the first 4 exons of *BCOR,* as with our case.

Given the rarity or this fusion in our study of additional tumors, and the fact that additional oncogenic alterations were detected in this case, we cannot exclude the possibility that the *BCOR-CREBBP* fusion represents a stochastic passenger event without meaningful oncogenic contribution. In particular, we recognize that one recent study suggested that most gene fusions detected by massively parallel sequencing are likely to be stochastic passenger events [18]. We also acknowledge that the majority of additional cases screened in this study are from adult patients. Given that CNS tumors with previously reported BCOR alterations, such as *BCOR* ex15 ITD, *EP300-BCOR* fusions, and loss-of-function mutations predominantly arise in pediatric or young adult patients, expanding analysis to larger cohorts enriched in pediatric patients would be warranted and may increase the chances of detecting further events involving *CREBBP* and/or *BCOR*.

The clinicopathological features of our index case are distinct from the previously reported pediatric gliomas with *EP300* and *BCOR* fusion events in several respects [46]. First, radiological features in our case showed a growth pattern consistent with “gliomatosis cerebri”, while the previously reported gliomas with *EP300-BCOR* fusions did not show this pattern to our knowledge. In addition, whereas the previous study demonstrated that cases with *EP300-BCOR* showed a myxoid to microcystic background, frequent calcifications, and sometimes piloid or even oligodendroglial-like features, these were not observed in our case which at initial biopsy demonstrated features of a classic infiltrating astrocytoma. While tumors in the prior *EP300-BCOR* series demonstrated rapid regrowth following resection, all patients were alive at the time of that publication (6 mo – 3.5 yrs. of available follow-up). In our case the patient demonstrated a relatively rapid disease course with high grade progression and treatment discontinuation 27 months following initial biopsy.

## Conclusion

We describe a rare *BCOR-CREBBP* fusion in a pediatric patient with a high-grade infiltrating astrocytoma who experienced progression and clinical deterioration within 27 months. In an additional 686 primary CNS tumor cases of adult and pediatric patients, assessed via FISH or RNA-seq analysis, we identified an additional case demonstrating a *BCOR* fusion to a paralog of *CREBBP*, namely *EP300*, similar to that seen in a recently reported series. In addition, we detected three additional fusions involving either *BCOR* or *CREBBP*, but with distinct partners. These findings add to the existing literature implicating *BCOR* as having a potential driving role in CNS tumors. However, given that the *BCOR-CREBBP* fusion here was not found to be recurrent and may represent a stochastic event, further screening and functional studies are warranted to further define the oncogenic potential of BCOR and related fusions in infiltrating gliomas.

## Supplementary information

**Additional file 1: Supplementary Table 1.** Single nucleotide variants (SNVs), amplifications, gene fusions, and indels from 161 unique genes covered by Oncomine Comprehensive Assay v3 (OCAv3) (ThermoFisher Scientific).

**Additional file 2: Supplementary Figure 1.** Immunohistochemical staining for BCOR. Infiltrating glioma cells in the index case are completely negative (a). A positive control on the same slide comprising an Ewing-like sarcoma with BCOR fusion demonstrate strongly positive nuclear labeling (b).

**Additional file 3: Supplementary Figure 2.** Comparison of chimeric transcripts generating from a *BCOR-CREBBP* fusion in the present case, BCOR-CREBBP and CREBBP-BCOR fusions in endometrial stromal sarcoma and EP300-BCOR fusions in pediatric glioma.

**Additional file 4: Supplementary Figure 3.** Gene set enrichment analysis of index case relative to 82 samples (67 patients) of distinct infiltrating gliomas. Shown are the enrichment scores for the two gene sets with a nominal *p* value (npv) and false discovery rate (FDR) of < 0.05, the gene set for HALLMARK_OXIDATIVE_PHOSPHORYLATION (OX_PHOS) and for HALLMARK_MYC_TARGETS_V1 (MYC_V1). Below each graph, the top ten genes with the highest enrichment scores are shown. For a complete list of genes in those two gene sets, please see the supplementary excel file 1 (Additional file 5). MYC_V1 npv = 0.0 and FDR = 0.00128; OX_PHOS npv = 0.0 and FDR = 0.00172.

**Additional file 5: Supplementary Excel File 1.** This file contains details for the full list of genes and corresponding enrichment scores for the genes comprising the OX_PHOS and MYC_V1 gene sets used in the gene set enrichment analysis.

## Data Availability

Data sharing is not applicable to this article.

## Abbreviations

CNS: Central nervous system
RNA-seq: RNA sequencing
BCOR: BCL6 interacting co-repressor
NGS: Next-generation sequencing
FISH: Fluorescence in-situ hybridization
TCGA: The Cancer Genome Atlas
MRI: Magnetic resonance imaging
PCGF1: Polycomb group ring finger 1
PUFD: PCGF Ub-like fold discriminator domain
PRC1.1: Polycomb repressive complex 1.1
HAT: Histone-acetyltransferase
ESS: Endometrial stromal sarcoma
