# Supplementary material for: Fusions involving BCOR and CREBBP are rare events in infiltrating glioma
Source: Acta Neuropathol Commun. 2020 Jun 3;8:80. doi: 10.1186/s40478-020-00951-4 (PMC7271411; doi:10.1186/s40478-020-00951-4)
Supplement: Supplementary file 1 — Additional file 1: Supplementary Table 1. Single nucleotide variants (SNVs), amplifications, gene fusions, and indels from 161 unique genes covered by Oncomine Comprehensive Assay v3 (OCAv3) (ThermoFisher Scientific). [file 40478_2020_951_MOESM1_ESM.pdf]

Supplementary Table S1. Genes interrogated by Oncomine panel

86 genes for the detection of hotspot mutations						
AKT1	*AKT2*	*AKT3*	*ALK*	*AR*	*ARAF*	*AXL*
BRAF	*BTK*	*CBL*	*CCND1*	*CDK4*	*CDK6*	*CHEK2*
CSF1R	*CTNNB1*	*DDR2*	*EGFR*	*ERB83*	*ERBB2*	*ERBB4*
ERCC2	*ESR1*	*EZH2*	*FGFR1*	*FGFR2*	*FGFR3*	*FGFR4*
FLT3	*FOXL2*	*GATA2*	*GNA11*	*GNAQ*	*GNAS*	*H3F3A*
HIST1H3B	*HNF1A*	*HRAS*	*IDH1*	*IDH2*	*JAK1*	*JAK2*
JAK3	*KIT*	*KNSTRN*	*KDR*	*KRAS*	*MAGOH*	*MAP2K1*
MAP2K2	*MAP2K4*	*MAPK1*	*MAX*	*MDM4*	*MED12*	*MET*
MTOR	*MYC*	*MYCN*	*MYD88*	*NFE2L2*	*NRAS*	*NTRK1*
NTRK2	*PDGFRA*	*PDGFRB*	*PIK3CA*	*PIK3CB*	*PPP2R1A*	*PTPN11*
RAC1	*RAF1*	*RET*	*RHEB*	*RHOA*	*ROS1*	*SF3B1*
SMAD4	*SMO*	*SPOP*	*SRC*	*STAT3*	*TERT*	*TOP1*
U2AF1	*XPO1*					

48 genes with full coding exonic regions for the detection of mutations						
ARID1A	*ATM*	*ATR*	*ATRX*	*BAP1*	*BRCA1*	*BRCA2*
CDK12	*CDKN1B*	*CDKN2A*	*CDKN2B*	*CHEK1*	*CREBBP*	*FANCA*
FANCD2	*FANCI*	*FBXW7*	*MLH1*	*MRE11A*	*MSH2*	*MSH6*
NBN	*NF1*	*NF2*	*NOTCH1*	*NOTCH2*	*NOTCH3*	*PALB2*
PIK3R1	*PMS2*	*POLE*	*PTCH1*	*PTEN*	*RAD51*	*RAD51B*
RAD51C	*RAD51D*	*RAD50*	*RB1*	*RNF43*	*SETD2*	*SLX4*
SMARCA4	*SMARCB1*	*STK11*	*TP53*	*TSC1*	*TSC2*	

47 genes for the detection of copy number alterations						
AKT1	*AKT2*	*AKT3*	*ALK*	*AR*	*AXL*	*BRAF*
CCND1	*CCND2*	*CCND3*	*CCNE1*	*CDK2*	*CDK4*	*CDK6*
CDKN2A	*CDKN2B*	*EGFR*	*ERBB2*	*ESR1*	*FGF19*	*FGF3*
FGFR1	*FGFR2*	*FGFR3*	*FGFR4*	*FLT3*	*IGF1R*	*KIT*
KRAS	*MDM2*	*MDM4*	*MET*	*MYC*	*MYCL*	*MYCN*
NTRK1	*NTRK2*	*NTRK3*	*PDGFRA*	*PDGFRB*	*PIK3CA*	*PIK3CB*
PPARG	*RICTOR*	*TERT*	*TSC1*	*TSC2*		

51 genes for the detection of gene fusions involving intergenic and intragenic regions						
AKT2	*ALK*	*AR*	*AXL*	*BRAF*	*BRCA1*	*BRCA2*
CDKN2A	*EGFR*	*ERB84*	*ERBB2*	*ERG*	*ESR1*	*ETV1*
ETV4	*ETVS*	*FGFR1*	*FGFR2*	*FGFR3*	*FGR*	*FLT3*
JAK2	*KRAS*	*MDM4*	*MET*	*MYB*	*MYBL1*	*NF1*
NOTCH1	*NOTCH4*	*NRG1*	*NTRK1*	*NTRK2*	*NTRK3*	*NUTM1*
PDGFRA	*PDGFRB*	*PIK3CA*	*PPARG*	*PRKACA*	*PRKACB*	*PTEN*
RAD51B	*RAF1*	*RB1*	*RELA*	*RET*	*ROS1*	*RSPO2*
RSPO3	*TERT*					
