# Supplementary material for: Fusions involving BCOR and CREBBP are rare events in infiltrating glioma
Source: Acta Neuropathol Commun. 2020 Jun 3;8:80. doi: 10.1186/s40478-020-00951-4 (PMC7271411; doi:10.1186/s40478-020-00951-4)
Supplement: Supplementary file 2 — Additional file 2: Supplementary Figure 1. Immunohistochemical staining for BCOR. Infiltrating glioma cells in the index case are completely negative (a). A positive control on the same slide comprising an Ewing-like sarcoma with BCOR fusion demonstrate strongly positive nuclear labeling (b). [file 40478_2020_951_MOESM2_ESM.pdf]

Supplementary Figure 1

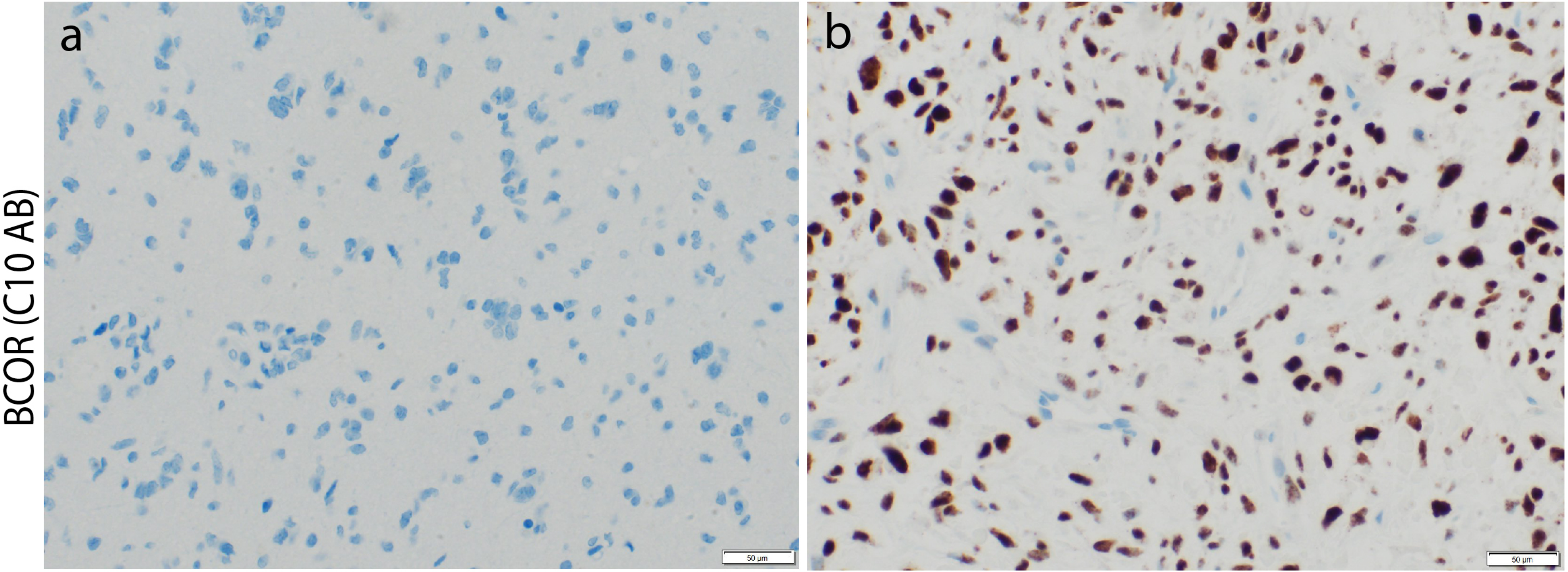

INDEX CASE - INFILTRATING GLIOMA

EWING-LIKE SARCOMA WITH BCOR FUSION
