# Supplementary material for: Fusions involving BCOR and CREBBP are rare events in infiltrating glioma
Source: Acta Neuropathol Commun. 2020 Jun 3;8:80. doi: 10.1186/s40478-020-00951-4 (PMC7271411; doi:10.1186/s40478-020-00951-4)

Supplementary Figure 2

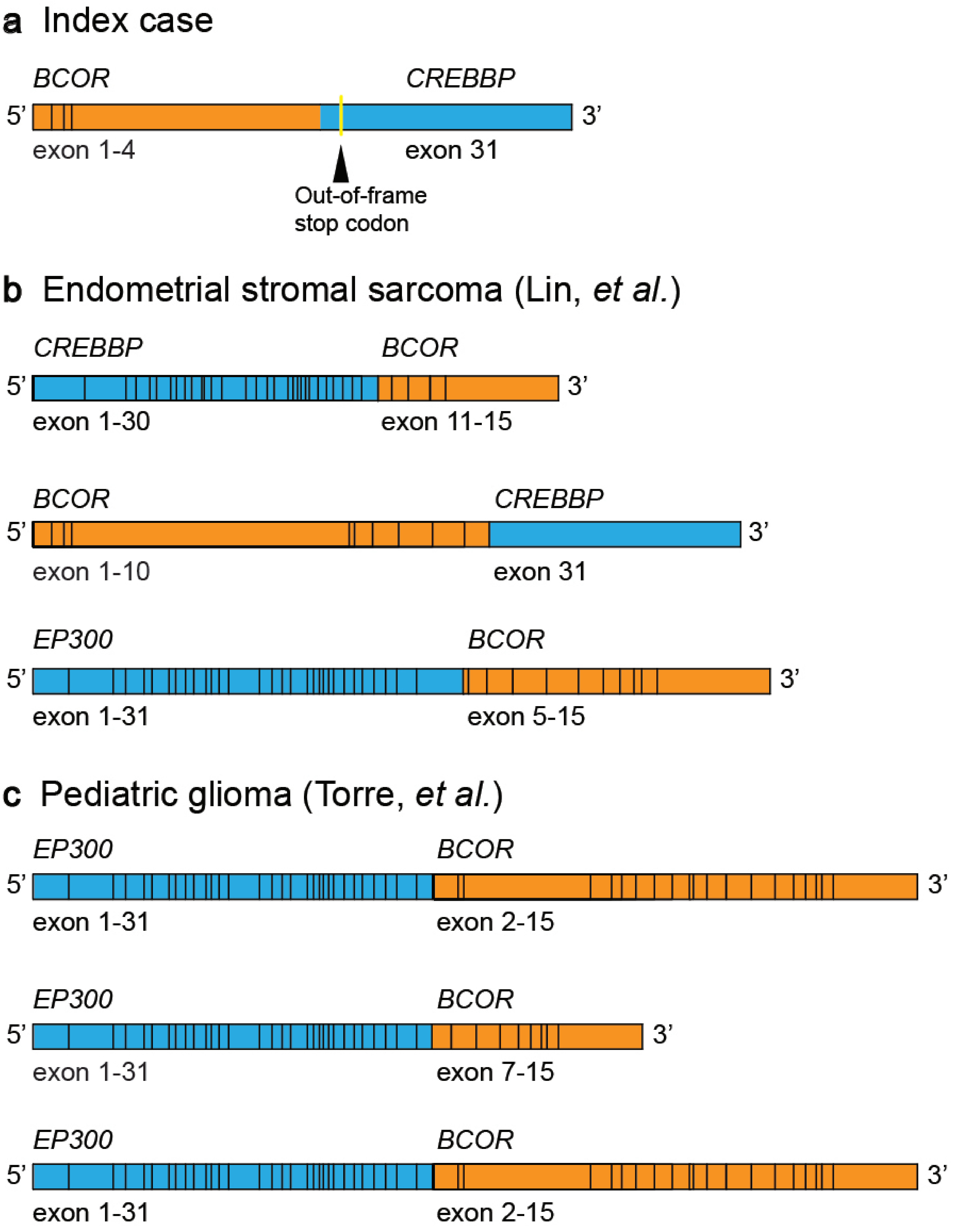

Supplement: Supplementary file 3 — Additional file 3: Supplementary Figure 2. Comparison of chimeric transcripts generating from a BCOR-CREBBP fusion in the present case, BCOR-CREBBP and CREBBP-BCOR fusions in endometrial stromal sarcoma and EP300-BCOR fusions in pediatric glioma. [file 40478_2020_951_MOESM3_ESM.pdf]
