# Supplementary material for: Fusions involving BCOR and CREBBP are rare events in infiltrating glioma
Source: Acta Neuropathol Commun. 2020 Jun 3;8:80. doi: 10.1186/s40478-020-00951-4 (PMC7271411; doi:10.1186/s40478-020-00951-4)
Supplement: Supplementary file 4 — Additional file 4: Supplementary Figure 3. Gene set enrichment analysis of index case relative to 82 samples (67 patients) of distinct infiltrating gliomas. Shown are the enrichment scores for the two gene sets with a nominal p value (npv) and false discovery rate (FDR) of < 0.05, the gene set for HALLMARK_OXIDATIVE_PHOSPHORYLATION (OX_PHOS) and for HALLMARK_MYC_TARGETS_V1 (MYC_V1). Below each graph, the top ten genes with the highest enrichment scores are shown. For a complete list of genes in those two gene sets, please see the supplementary excel file 1 (Additional file 5). MYC_V1 npv = 0.0 and FDR = 0.00128; OX_PHOS npv = 0.0 and FDR = 0.00172. [file 40478_2020_951_MOESM4_ESM.pdf]

Supplementary Figure 3

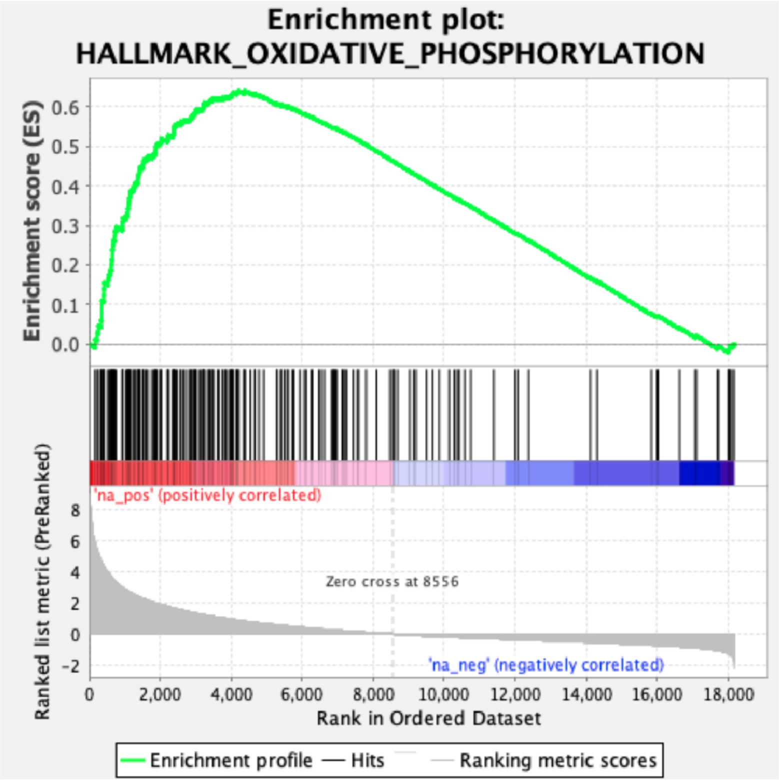

RANK IN GENE SET	GENE	OVERALL RANK
1	BDH2	166
2	COX7C	224
3	PHB2	260
4	NDUFS4	333
5	NDUFC1	350
6	LDHB	351
7	TIMM9	352
8	NDUFB8	409
9	UQCRH	412
10	OAT	423

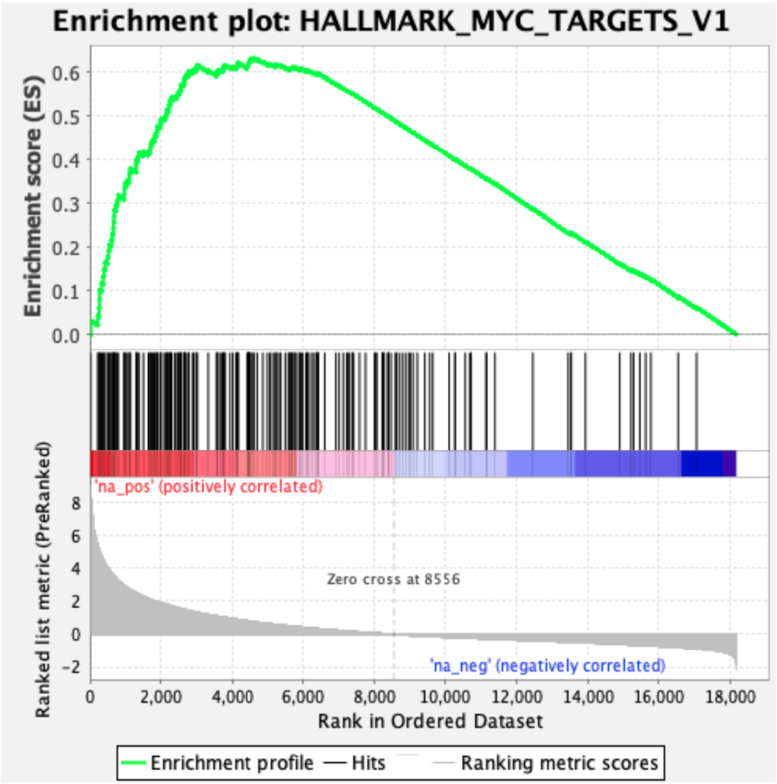

RANK IN GENE SET	GENE	OVERALL RANK
1	RPL34	30
2	HNRNPA3	221
3	PHB2	260
4	RPL6	270
5	RPL22	274
6	RPS3	330
7	RPS6	377
8	HDDC2	395
9	U2AF1	432
10	SNRPD2	494
